# Immune Thrombocytopenia Induced by Immune Checkpoint Inhibitors in Solid Cancer: Case Report and Literature Review

**DOI:** 10.3389/fonc.2020.530478

**Published:** 2020-12-07

**Authors:** Xiaolin Liu, Xiuju Liang, Jing Liang, Yan Li, Jun Wang

**Affiliations:** ^1^ Department of Oncology, The First Affiliated Hospital of Shandong First Medical University, Jinan, China; ^2^ Department of Oncology, No. 960 Hospital, The People’s Liberation Army, Jinan, China

**Keywords:** programmed cell death 1 inhibitor, programmed cell death ligand 1, immune thrombocytopenia, immune checkpoint inhibitor, durvalumab, immune-related adverse event

## Abstract

Immune checkpoint inhibitors, including antibodies targeting programmed cell death protein-1 (PD-1) and its receptor programmed cell death ligand-1 (PD-L1), represent promising therapeutic strategies for advanced human malignancies. However, a subgroup of patients experiences various autoimmune toxicities, termed immune-related adverse events (irAEs), that occur as a result of on-target and off-tumor autoimmune responses. Although irAEs are generally confirmed to be less severe than toxicities caused by conventional chemotherapy and targeted therapy, uncommon irAEs, such as immune thrombocytopenia, may occur with a very low incidence and sometimes be severe or fatal. This review focuses on the epidemiology, clinical presentation, and prognosis of immune thrombocytopenia occurring in advanced cancer patients induced by immune checkpoint inhibitors, especially in those with PD-1 or PD-L1 inhibitor treatment. We also first present one patient with non-small cell lung cancer who received the PD-L1 inhibitor durvalumab and developed severe thrombocytopenia.

## Introduction

Immune checkpoint inhibitors (ICIs) are intended to destroy tumor cells by disrupting the immunoinhibitory signals mediated by programmed cell death protein-1 (PD-1), programmed cell death ligand-1 (PD-L1), and cytotoxic T-lymphocyte-associated protein 4 (CTLA-4) and unleash the power of the body’s immune system (1). In particular, immunotherapy with anti-PD-1/PD-L1 antibodies has resulted in durable tumor remission and changed the treatment landscape in non-small cell lung cancer (NSCLC) and melanoma, and the application of PD-1/PD-L1 inhibitors is expending to various other human tumors (2).

ICIs are generally recognized as tolerable agents that are associated with unique side effects termed immune-related adverse effects (irAEs), and these toxicities may concern any organ or tissue. However, irAEs present in nearly 50% of patients treated with PD-1/PD-L1 inhibitors, and 10%–15% of patients are likely to develop grade 3–4 or potentially life-threatening events with variable clinical implications, leading to a discontinuation of immunotherapy. Fatigue, loss of appetite, rash, pruritis, pneumonitis, hepatitis, and hypothyroidism are identified as usual irAEs (3). Use of PD-1/PD-L1 inhibitors also results in a variety of irAEs at a low frequency, such as eye, cardiac, neurological, and hematologic toxicities. Hematologic disorders include aplastic anemia, neutropenia, thrombocytopenia, bi-cytopenia, hypereosinophilia, and pancytopenia. Generally, hematologic complications are much less frequently found in patients treated with anti-PD-1/PD-L1 therapy compared to those induced with classic cytotoxic chemotherapy, but some are potentially life threatening. An increased number of cases of hematologic disorders have been reported since 2017, which may be attributed to increased application of ICIs and improved recognition of adverse effects (4). In particular, immune thrombocytopenia induced by ICIs has been reported in a few NSCLC, melanoma, and other malignancies (5–25). We herein describe one case of immune thrombocytopenia induced by the PD-L1 inhibitor durvalumab and its clinical management. We also review the epidemiology, clinical presentation, and prognosis of immune thrombocytopenia occurring in advanced cancer patients caused by ICIs.

## Case Presentation

**Figure 1:**
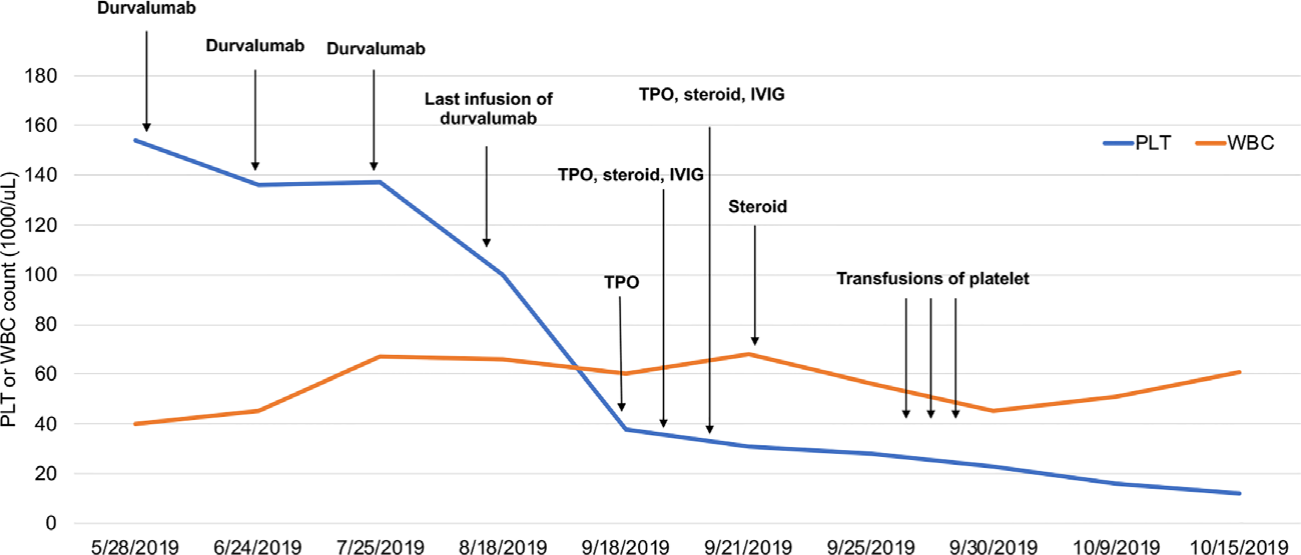
Platelet and WBC counts following immunotherapy with durvalumab. WBCs are multiplied by 10^2^.

In July 2018, an 82-year-old woman was referred to our hospital. Her medical history was not remarkable. A computed tomography (CT) scan of the chest revealed a mass in her left upper lobe with mediastinum lymph node metastasis and left pleural effusion. She was initially diagnosed with lung adenocarcinoma by fiberoptic bronchoscopy. Molecular mutation analysis showed that her tumor did not harbor any driver gene alterations, such as EGFR, ALK, and ROS1. Immunohistochemical staining of tumor tissue showed that PD-L1 expression was found in >25% of tumor cells. In August 2018, she entered a clinical study and received her first infusion of the anti-PD-L1 antibody durvalumab as first-line therapy. At that time, baseline blood tests were normal. After 4 infusions of durvalumab, she got a partial response by a CT scan and no severe toxicities in October 2018, and immunotherapy was continued. Shortly after receiving 20 cycles of durvalumab treatment, this patient developed thrombocytopenia (platelet level: 100 × 10^3^/μL) with normal hemoglobin and normal white cell counts. Unfortunately, 2 weeks later, her thrombocytopenia worsened with a sudden decrease to 38 × 10^3^/μL platelet level. A bone narrow biopsy was performed on October 20, 2018, and showed no dysplastic changes. Abnormality of lymphoid and megakaryocytic cell lines were not found, and evidence of metastatic cancer invasion was not present. Antinuclear and antiplatelet antibodies were not abnormal. Other laboratory tests were normal or negative. After excluding chemotherapy, infectious etiology, or other drug-induced thrombocytopenia, we considered a diagnosis of immune thrombocytopenia induced by durvalumab. She received a high-dose steroid (1 mg/kg) for 4 consecutive days and was treated with recombinant human thrombopoietin (TPO), but platelets did not recover. She received two platelet transfusions, but the platelet count was not increased. The lowest platelet level was 12 × 10^3^/μL on October 15, 2019. Thus, immunotherapy with durvalumab was definitely discontinued because of unacceptable toxicities. Subsequent treatments, including continuing platelet transfusions and infusion of intravenous immunoglobulin (IVIG), were not effective (**Figure 1**). Finally, this patient unfortunately died of acute upper gastrointestinal hemorrhage.

## Incidence and Onset of Immune Thrombocytopenia Induced by ICIs

Fatigue, loss of appetite, rash, pruritis, pneumonitis, hepatitis, and hypothyroidism are identified as usual irAEs. However, hematologic disorders are uncommon. Rare cases of leukopenia, thrombocytopenia, bi-cytopenia, hypereosinophilia, and pancytopenia have been reported. Basically, thrombocytopenia was reported as an independent hematologic adverse event. Recently, a NSCLC patient who developed concomitant immune-related thrombocytopenia and hypothyroidism following nivolumab treatment was also reported (5).

Although ICIs have been approved for treating various types of human malignancies, immune-related thrombocytopenia is most likely to be reported in NSCLC (5–11, 24, 25), melanoma (12–20), lymphoma (21), pancreatic cancer (22), and renal cell carcinoma (23), which is consistent with retrospective data from multiple institutions (26). Previous case and retrospective studies show that immune thrombocytopenia occurred in cancer patients treated with PD-1 inhibitor monotherapy, such as nivolumab or pembrolizumab, or a CTLA-4 inhibitor, or a combination with PD-1 and a CTLA-4 inhibitor (**Table 1**). Here, we report the first case of a lung cancer patient who developed a late-onset autoimmune cause of thrombocytopenia during PD-L1 inhibitor durvalumab monotherapy.

**Table 1:** Summary of reported cases with immune-related thrombocytopenia after receiving immunotherapy.

Authors	Year		Tumor type		Age (years)	Gender	ICI	Cycles	Lowest PLT (/ul)	Concurrent irAE	PA-IgG (ng/10^7^ cells)	irAE Treatment	BRT	Final outcome
**Lung cancer**														
Present case		2019		LAC	82	Female	D	12	12000	None	NR	Steroid, IVIG	PR	Died of irAE
Mouri et al. (24)		2020		LAC	66	Male	P	1	3000	None	154	Steroid	PR	Recovered
Dickey et al. (25)		2020		LSCC	60	Female	P	5	77000	Anemia	NR	Steroid	PD	Recovered
Hasegawa et al. (6)		2019		LAC	82	Female	N	2	2000	None	223	Steroid, IVIG, TRA	NR	Died of irAE
Song et al. (7)		2019		NSCLC	65	Male	P	7	0	None	NR	Steroid, IVIG, TRA	SD	NR
Mori et al. (8)		2019		NSCLC	77	Male	N	1	2000	Pneumonitis	1130	Steroid	SD	Recovered
Tokumo et al. (11)		2018		LAC	56	Male	N	3	19000	Pancytopenia	NR	Steroid, IVIG	PR	Died of Cancer
Jotatsu et al. (5)		2017		NSCLC	62	Male	N	2	1600	Hypothyroidism	473	Steroid	PR	Recovered
Karakas et al. (9)		2017		NSCLC	78	Male	N	6	5000	None	NR	Steroid	NR	Died of Cancer
Bagley et al. (10)		2016		LSCC	34	Male	N	8	33000	None	NR	TRA	PR	Recovered
**Melanoma**														
Berger et al. (12)	2019		Melanoma		57	Female	P	11	10000	Anemia	NR	Steroid, CTX, IVIG	PD	Died of Cancer
Sadaat et al. (13)	2018		Melanoma		58	Male	P	6	10100	HLH	NR	Steroid	PR	Recovered
Philipp et al. (14)	2018		Melanoma		46	Female	N, I	2	7000	Neutropenia	NR	Steroid, IVIG	CR	Recovered
Takimoto et al. (15)	2018		Melanoma		79	Female	N, I	2	2000	None	NR	Steroid, IVIG, TRA	CR	Recovered
Shiuan et al. (16)	2017		Melanoma		47	Female	N, I	1	5000	None		Steroid, IVIG, rituximab, TRA	PR	Recovered
Shiuan et al. (16)	2017		Melanoma		45	Female	N, I	1	75000	None	NR	Steroid, IVIG, rituximab	PD	NR
Pföhler et al. (20)	2017		Melanoma		73	Male	P	1	10800	None	NR	Steroid	PD	Died of Cancer
Inadomi et al. (17)	2016		Melanoma		73	Male	N	7	23000	Bi-cytopenia	28.4	Steroid	PD	Died of Cancer
Kanameishi et al. (18)	2016		Melanoma		75	Female	N	2	2000	None	NR	Steroid, IVIG, TRA	NR	Recovered
Le Roy et al. (19)	2016		Melanoma		34	Female	P	1	100	None	NR	Steroid, IVIG	NR	Recovered
Le Roy et al (19)	2016		Melanoma		51	Female	P	9	9000	None	NR	Steroid	CR	Recovered
**Others**														
Iyama et al. (22)	2020		Pancreatic cancer		54	Female	N	2	12000	None	Slightly elevated	Steroid, IVIG, CSA	NR	Recovered
Hata et al. (23)	2020		RCC		70	Male	N	8	17000	None	73	Steroid, IVIG, TRA	NR	Died of Cancer
Bulbul et al. (21)	2017		HL		32	Female	N	15	29000	Leucopenia	ND	Steroid, IVIG	CR	Recovered

LAC, lung adenocarcinoma; NSCLC, non-small cell lung cancer; LSCC, lung squamous cell carcinoma; RCC, renal cell carcinoma; HL, Hodgkin’s lymphoma; ICI, immune checkpoint inhibitor; D, durvalumab; N, nivolumab; P, pembrolizumab; I, ipilimumab; irAE, immune-related adverse event; HLH, Hemophagocytic Lymphohistiocytosis; NR, not reported; ND, not detected; IVIG, intravenous immunoglobulin; TRA, thrombopoietin receptor agonist; CTX, cyclophosphamide; BRT, best response to treatment; PR, partial response; CR, complete response; SD, stable disease; PD, progressive disease; CSA, cyclosporine.

The absolute frequency of hematologic irAEs, including immune thrombocytopenia, is unknown. The frequency of thrombocytopenia in clinical phase II–III trials involving nivolumab monotherapy or combined with brentuximab is between 6% and 13% for all grades and between 1% and 10% for grades 3 to 4 (27–29). Phase II–III trials seem to report a higher frequency of immune thrombocytopenia than retrospective studies involving multiple institutions or large data sets. For example, Shiuan et al. reported nearly 0.5% of metastatic melanoma patients experienced immune thrombocytopenia by reviewing a total of 2360 patients with melanoma receiving checkpoint inhibitor therapy from five large institutions in the United States (16). Data from a descriptive observational study including cancer patients registered in three French pharmacovigilance databases showed that PD-1/PD-L1 inhibitor-induced hematological irAEs had a frequency of 0.5%. Of them, thrombocytopenia was also the most common type of hematological irAEs (26). Davis et al. identified 168 cases of immune thrombocytopenic purpura complicating ICI therapy in individual case safety reports using the World Health Organization’s pharmacovigilance database VigiBase (4). Immune thrombocytopenic purpura was the most common reported hematologic toxicity. However, VigiBase does not capture the number of individuals actually exposed to a given ICI, so the absolute frequency of hematologic toxicities cannot be calculated. In a meta-analysis conducted by Wang et al., the relative risks of all- and high-grade thrombocytopenia were lower in patients who received ICIs than those in control therapies (30). A low incidence of high-grade thrombocytopenia was observed in cancer patients treated with PD-1 inhibitor monotherapy compared to those with PD-1 inhibitor combination therapy (31). Combined analysis showed that only 2% of patients developed all-grade thrombocytopenia (95% CI: 1%–5%), but the use of a PD-1/PD-L1 inhibitor in combination therapy with ipilimumab, peptide vaccines, or chemotherapy had significantly higher risks than PD-1 inhibitor monotherapy (6%, 95% CI: 2%–18%) (30). However, the addition of pembrolizumab to chemotherapy failed to improve the risk of thrombocytopenia with an incidence of 5% for combination therapy in the randomized KEYNOTE-021 study (32). Although hematologic disorders have been found in diverse tumor types, the incidence rate and severity of all-grade thrombocytopenia, leukopenia, and neutropenia appeared to be similar in NSCLC and melanoma, which was reported in a meta-analysis (30).

Furthermore, immune thrombocytopenia is an unusual irAE, but deaths caused by this severe adverse event were reported. In a recently published meta-analysis involving 125 clinical trials and 20,128 patients, a total of 82 deaths (0.45%) were reviewed. Thrombocytopenic (1.2%) or immune thrombocytopenic purpura (1.2%) were identified as uncommon causes of treatment-related death (33). In VigiBase, death was reported in 11% of immune thrombocytopenia cases (4). Thus, immune thrombocytopenia is a potentially life-threatening side effect and should not be neglected in clinical practice.

Time of onset of development of immune thrombocytopenia is not clear. The risk for developing irAEs was usually highest during the initial 4 weeks of immunotherapy, but irAEs occurred in 5.7% of patients in the period from 4 weeks to the end of treatment as late-onset toxicity even in patients whose immunotherapy was terminated (34, 35). The median time to onset of hematological irAEs is similar to times for other irAEs. In a majority of reported cases, thrombocytopenia occurred as early as initial 1–2 cycles of treatment (5). Our and other case reports showed that it also could develop as a delayed toxicity in which thrombocytopenia happened after approximately 1–2 years of treatment (12, 21). Data from the VigiBase showed that immune thrombocytopenic purpura may develop at any time point with a median of 41 days (range from 14 to 321 days) (4).

## Diagnosis and Treatment of Immune Thrombocytopenia Induced by ICIs

Diagnosis of immune thrombocytopenia is difficult and challenging due to the lack of a specific test or marker. Although immune-related thrombocytopenia could be induced by ICIs, other secondary causes may lead to this disease, including infection and other therapeutic agents. The diagnosis depends on the exclusion of other causes of a low platelet count; additional investigations, such as bone marrow examination, may be necessary in some cases to exclude dysplasia or cancer invasion. In particular, when the irAEs concern platelets, it should be distinguished from a chemotherapy-induced thrombocytopenia for patients receiving chemotherapy plus immunotherapy because the prognosis and therapeutic management differs. Sometimes detecting the presence of a high platelet–associated IgG titer is helpful to confirm immune thrombocytopenia (8). Increased circulating PD-1+CD4+T cells and PD-L1+DCs and sPD-1 levels, which has been reported in idiopathic thrombocytopenic purpura, may be helpful in identifying immune-related thrombocytopenia (36, 37).

Approved treatments for immune thrombocytopenia are uncertain. Different clinical guidelines on the management of irAEs have been published by the European Society of Medical Oncology, the Society for Immunotherapy of Cancer, and the American Society of Clinical Oncology (38–40). Any new blood count abnormality, such as decreased platelet counts, should be considered as a potentially clinically significant event related to immunotherapy because the early onset of hematological toxicities developed as a grade 1 or 2 event could worsen within a short period. Similar to our case, platelet count was slightly decreased or only close to the lower limit of normal values (100 × 10^3^/μL) before or during ICI therapy. It is possible that immunotherapy with ICIs aggravates thrombocytopenia *via* autoimmune activation, especially for elderly cancer patients.

Basically, regular management includes the use of recombinant human TPO or romiplostim, a recombinant TPO receptor agonist (TRA), and platelet transfusion. Other treatment strategies, such as IVIG, steroids (methylprednisolone pulse with maintenance therapy), splenectomy, using other immunosuppressive agents such as azathioprine and rituximab, were required. Of these interventions, steroids were the most frequently used agent for treating mild thrombocytopenia, but they are not always effective in managing immune-related severe thrombocytopenia caused by ICIs. Previous investigations indicate that idiopathic thrombocytopenic purpura patients with the HLA-DRB1*0410 or HLA-DRB1*0405 allele were originally resistant to steroid treatment (6), which needs to be validated in immune thrombocytopenia secondary to ICIs. Accordingly, these patients who are resistant to steroid treatment could timely switch to other treatments, such as IVIG, rituximab, and a TRA (16). On the other hand, the predictive value of irAEs caused by immunotherapy have been evaluated by a variety of retrospective studies (41–44). Objective response rate and survival were significantly improved in patients who experienced different irAEs compared with those who did not experienced them. Unlike other common specific irAEs (45), hematologic events, such as thrombocytopenia, has not been found to be linked to increased efficacy of immunotherapy, but half of the previously reported cases with immune thrombocytopenia responded well to immunotherapy (**Table 1**). In the present case report, this patient had a deep disease response beyond 12 months of immunotherapy. Thus, monitoring symptoms of bleeding and the blood cell count during and after any immunotherapy is very important to help recognize and identify patients at risk of bleeding early, especially those whose tumors were responsive to immunotherapy, and rapidly interfere with steroids and other agents to obtain consistent benefits and good outcomes.

## Mechanism of Immune Thrombocytopenia Induced by ICIs

Although at least six different mechanisms of drug-induced thrombocytopenia have been proposed, the mechanism underlying thrombocytopenia induced by immune checkpoint blockade remains unclear (46). It is reasonable that the activation of the body’s immune system contributes to immune-related thrombocytopenia and other hematologic disorders. The activation of CD4+ helper T cells and CD8+ cytotoxic T cells is involved in the immune response in patients receiving CTLA-4 or PD-1/PD-L1 inhibitors, resulting in the damage to hematopoietic stem cells (47). Furthermore, a circulating immune response may contribute to a decreased thrombocyte count. In NSCLC cases, nivolumab induced or increased production of platelet-specific IgG autoantibodies that could promote platelet destruction with immature platelets and megakaryocytes without abnormal cells in a bone marrow biopsy (5, 8). The pathogenesis of thrombocytopenia induced by ICIs is postulated to be similar to classical immune thrombocytopenia, including idiopathic thrombocytopenic purpura. For example, Wu et al. found that the levels of interferon-γ, interleukin-17, and sPD-1 in the serum of patients with idiopathic thrombocytopenic purpura were increased, and IL-4 and transforming growth factor-β were decreased. Furthermore, activation of PD-1/PD-L1 signaling with sPD-L1 may restore the imbalance of Th1/Th2 and Treg/Th17 cell subtypes, which could be a therapeutic strategy for idiopathic thrombocytopenic purpura or immune thrombocytopenia (36).

## Conclusions

Autoimmune hematologic toxicities induced by ICIs, including thrombocytopenia, are viewed as rare irAEs, and increased application of ICIs in advanced malignancies contributes to increased reports of immune thrombocytopenia, but it should not be neglected in treating patients with ICIs because it is potentially life threatening in some cases. Oncologists should bear in mind that decreasing platelet counts represent an early sign of immune-related thrombocytopenia. In patients with immune thrombocytopenia, the risk of bleeding, arterial thromboembolism, or venous thrombosis is increased. Careful recognition, diagnosis, and differential diagnosis are required. Clinical management includes the use of steroid, IVIG, and platelet transfusion. However, the true mechanism of immunotherapy-related thrombocytopenia and its pathogenesis is unknown and further investigation is awaited.

## Ethics Statement

Written informed consent was obtained from the participant for the publication of this case report and any potentially identifying clinical information.

## Author Contributions

XLL was involved in the identification and selection of patient cases and drafted the manuscript. XJL and JL were involved in the drafting and editing of the manuscript. YL reviewed and edited the manuscript. JW was involved in the identification, selection, and management of patient cases and reviewed and edited the manuscript. All authors contributed to the article and approved the submitted version.

## Funding

This study was supported by National Natural Science Foundation of China (Grant No. 81572875). CSCO-MSD Cancer Research Foundation (Grant No. Y-MSD2020-0350), and CSCO-PILOT Cancer Research Foundation (Grant No. Y-2019AZMS-0440).

## Conflict of Interest

The authors declare that the research was conducted in the absence of any commercial or financial relationships that could be construed as a potential conflict of interest.

## Abbreviations

ALK, Anaplastic lymphoma kinase translocations; CT, Computerized tomography; CTLA-4, Cytotoxic T-lymphocyte-associated protein 4; EGFR, Endothelial growth factor receptor; FDA, United States Food and Drug Administration; irAEs, Immune-related adverse events; ICI, Immune checkpoint inhibitor; IVIG, Intravenous immunoglobulin; NSCLC, Non-small cell lung cancer; PD-1, Programmed cell death protein-1; PD-L1, Programmed death ligand-1; TPO, thrombopoietin; TRA, thrombopoietin receptor agonist.

## References

[1] Schreiber RD Old LJ Smyth MJ Cancer immunoediting: integrating immunity’s roles in cancer suppression and promotion. Science (2011) 331(6024):1565–70. 10.1126/science.1203486 21436444

[2] Postow MA Callahan MK Wolchok JD Immune checkpoint blockade in cancer therapy. J Clin Oncol (2015) 33(17):1974–82. 10.1200/JCO.2014.59.4358 PMC4980573 25605845

[3] Naidoo J Page DB Li BT Connell LC Schindler K Lacouture ME Toxicities of the anti-PD-1 and anti-PD-L1 immune checkpoint antibodies. Ann Oncol (2016) 27(7):1362. 10.1093/annonc/mdw141 27072927 PMC5006114

[4] Davis EJ Salem JE Young A Green JR Ferrell PB Ancell KK Hematologic complications of immune checkpoint inhibitors. Oncologist (2019) 24(5):584–8. 10.1634/theoncologist.2018-0574 PMC6516131 30819785

[5] Jotatsu T Oda K Yamaguchi Y Noguchi S Kawanami T Kido T Immune-mediated thrombocytopenia and hypothyroidism in a lung cancer patient treated with nivolumab. Immunotherapy (2018) 10(2):85–91. 10.2217/imt-2017-0100 29260625

[6] Hasegawa T Ozaki Y Inoue T Watanabe Y Fukuhara M Yamaura T Nivolumab-related severe thrombocytopenia in a patient with relapsed lung adenocarcinoma: a case report and review of the literature. J Med Case Rep (2019) 13(1):316. 10.1186/s13256-019-2245-y 31647029 PMC6813076

[7] Song P Zhang L Eltrombopag treatment for severe refractory thrombocytopenia caused by pembrolizumab. Eur J Cancer (2019) 121:4–6. 10.1016/j.ejca.2019.08.003 31522129

[8] Mori H Sakai C Iwai M Sasaki Y Gomyo T Toyoshi S Immune thrombocytopenia induced by nivolumab in a patient with non-small cell lung cancer. Respir Med Case Rep (2019) 28:100871. 10.1016/j.rmcr.2019.100871 31198679 PMC6557745

[9] Karakas Y Yuce D Kilickap S Immune thrombocytopenia induced by nivolumab in a metastatic non-small cell lung cancer patient. Oncol Res Treat (2017) 40(10):621–2. 10.1159/000477968 28950270

[10] Bagley SJ Kosteva JA Evans TK Langer CJ Immune thrombocytopenia exacerbated by nivolumab in a patient with non-small-cell lung cancer. Can Treat Commun (2016) 6:20–3. 10.1016/j.ctrc.2016.02.009

[11] Tokumo K Masuda T Miyama T Miura S Yamaguchi K Sakamoto S Nivolumab-induced severe pancytopenia in a patient with lung adenocarcinoma. Lung Cancer (2018) 119:21–4. 10.1016/j.lungcan.2018.02.018 29656748

[12] Berger M Amini-Adle M Crumbach L Paubelle E Dalle S A case of immune thrombocytopaenia induced by pembrolizumab in a metastatic melanoma patient with a history of immune-mediated pure red cell aplasia. Eur J Cancer (2019) 112:94–7. 10.1016/j.ejca.2019.02.006 30954716

[13] Sadaat M Jang S Hemophagocytic lymphohistiocytosis with immunotherapy: brief review and case report. J Immunother Cancer (2018) 6(1):49. 10.1186/s40425-018-0365-3 29871698 PMC5989389

[14] Philipp M Frischhut N Tschachler A Steinkohl F Weinlich G Schmuth M Pseudoprogression with subsequent complete response and severe thrombocytopenia to checkpoint inhibitor immunotherapy in a patient with advanced mucosal melanoma of the sinonasal cavity. Anticancer Drugs (2018) 29(9):914–8. 10.1097/CAD.0000000000000664 29952773

[15] Takimoto R Otsuka A Kaku Y Honda T Kabashima K No recurrence of nivolumab-induced idiopathic thrombocytopenic purpura in a metastatic melanoma patient switched to ipilimumab. Eur J Dermatol (2018) 28(1):84–5. 10.1684/ejd.2017.3151 29400292

[16] Shiuan E Beckermann KE Ozgun A Kelly C McKean M McQuade J Thrombocytopenia in patients with melanoma receiving immune checkpoint inhibitor therapy. J Immunother Cancer (2017) 5:8. 10.1186/s40425-017-0210-0 28239462 PMC5319013

[17] Inadomi K Kumagai H Arita S Tsuruta N Takayoshi K Mishima K Bi-cytopenia possibly induced by anti-PD-1 antibody for primary malignant melanoma of the esophagus: A case report. Medicine (Baltimore) (2016) 95(29):e4283. 10.1097/MD.0000000000004283 27442668 PMC5265785

[18] Kanameishi S Otsuka A Nonomura Y Fujisawa A Endo Y Kabashima K Idiopathic thrombocytopenic purpura induced by nivolumab in a metastatic melanoma patient with elevated PD-1 expression on B cells. Ann Oncol (2016) 27(3):546–7. 10.1093/annonc/mdv580 26602778

[19] Le Roy A Kempf E Ackermann F Routier E Robert C Turpin A Two cases of immune thrombocytopenia associated with pembrolizumab. Eur J Cancer (2016) 54:172–4. 10.1016/j.ejca.2015.10.073 26687374

[20] Pfohler C Eichler H Burgard B Krecké N Müller CSL Vogt T A Case of Immune Thrombocytopenia as a Rare Side Effect of an Immunotherapy with PD1-Blocking Agents for Metastatic Melanoma. Transfus Med Hemother (2017) 44(6):426–8. 10.1159/000479237 PMC5757577 29344020

[21] Bulbul A Mustafa A Chouial S Rashad S Idiopathic thrombocytopenic purpura and autoimmune neutropenia induced by prolonged use of nivolumab in Hodgkin’s lymphoma. Ann Oncol (2017) 28(7):1675–6. 10.1093/annonc/mdx159 28379290

[22] Iyama S Takada K Yoshida M Takahashi D Kobune M Acquired amegakaryocytic thrombocytopenic purpura possibly induced by anti-PD-1 antibody. Ann Hematol (2020) 99(7):1669–70. 10.1007/s00277-020-04053-y 32367179

[23] Hata S Abe S Severe immune thrombocytopenia induced by nivolumab in a patient with metastatic renal cell carcinoma. Urol Case Rep (2020) 32:101128. 10.1016/j.eucr.2020.101128 32489885 PMC7256289

[24] Mouri A Kaira K Shiono A Miura YU Kagamu H Thrombocytopenia Associated With Pembrolizumab in Patients With Non-small Cell Lung Cancer (NSCLC): A Case Report and Literature Review. In Vivo (2020) 34(2):877–80. 10.21873/invivo.11852 PMC7157878 32111798

[25] Dickey MS Raina AJ Gilbar PJ Wisniowski BL Collins JT Karki B Pembrolizumab-induced thrombotic thrombocytopenic purpura. J Oncol Pharm Pract (2020) 26(5):1237–40. 10.1177/1078155219887212 31718453

[26] Delanoy N Michot JM Comont T Kramkimel N Lazarovici J Dupont R Haematological immune-related adverse events induced by anti-PD-1 or anti-PD-L1 immunotherapy: a descriptive observational study. Lancet Haematol (2019) 6(1):e48–57. 10.1016/S2352-3026(18)30175-3 30528137

[27] Weber JS D’Angelo SP Minor D Hodi FS Gutzmer R Neyns B Nivolumab versus chemotherapy in patients with advanced melanoma who progressed after anti-CTLA-4 treatment (CheckMate 037): a randomised, controlled, open-label, phase 3 trial. Lancet Oncol (2015) 16(4):375–84. 10.1016/S1470-2045(15)70076-8 25795410

[28] Sharma P Callahan MK Bono P Nivolumab monotherapy in recurrent metastatic urothelial carcinoma (CheckMate 032): a multicentre, open-label, two-stage, multi-arm, phase 1/2 trial. Lancet Oncol (2016) 17(11):1590–8. 10.1016/S1470-2045(16)30496-X PMC5648054 27733243

[29] Zinzani PL Santoro A Gritti G Brice P Barr PM Kuruvilla J Nivolumab Combined With Brentuximab Vedotin for Relapsed/Refractory Primary Mediastinal Large B-Cell Lymphoma: Efficacy and Safety From the Phase II CheckMate 436 Study. J Clin Oncol (2019) 37(33):3081–9. 10.1200/JCO.19.01492 PMC6864847 31398081

[30] Wang S Hao J Wang H Fang Y Tan L Efficacy and safety of immune checkpoint inhibitors in non-small cell lung cancer. Oncoimmunology (2018) 7(8):e1457600. 10.1080/2162402X.2018.1457600 30221052 PMC6136858

[31] Sui JD Wang Y Wan Y Wu YZ Risk of hematologic toxicities with programmed cell death-1 inhibitors in cancer patients: a meta-analysis of current studies. Drug Des Devel Ther (2018) 12:1645–57. 10.2147/DDDT.S167077 PMC5996859 29922039

[32] Langer CJ Gadgeel SM Borghaei H Papadimitrakopoulou VA Patnaik A Powell SF Carboplatin and pemetrexed with or without pembrolizumab for advanced, non-squamous non-small-cell lung cancer: a randomised, phase 2 cohort of the open-label KEYNOTE-021 study. Lancet Oncol (2016) 17(11):1497–508. 10.1016/S1470-2045(16)30498-3 PMC6886237 27745820

[33] Wang Y Zhou S Yang F Qi X Wang X Guan X Treatment-related adverse events of pd-1 and pd-l1 inhibitors in clinical trials: a systematic review and meta-analysis. JAMA Oncol (2019) 5(7):1008–19. 10.1001/jamaoncol.2019.0393 PMC6487913 31021376

[34] Kanjanapan Y Day D Butler MO Wang L Joshua AM Hogg D Delayed immune-related adverse events in assessment for dose-limiting toxicity in early phase immunotherapy trials. Eur J Cancer (2019) 107:1–7. 10.1016/j.ejca.2018.10.017 30529898

[35] Wang LL Patel G Chiesa-Fuxench ZC McGettigan S Schuchter L Mitchell TC Timing of onset of adverse cutaneous reactions associated with programmed cell death protein 1 inhibitor therapy. JAMA Dermatol (2018) 154(9):1057–61. 10.1001/jamadermatol.2018.1912 PMC6143042 30027278

[36] Wu D Liu Y Pang N Sun M Wang X Haridia Y PD-1/PD-L1 pathway activation restores the imbalance of Th1/Th2 and treg/Th17 cells subtypes in immune thrombocytopenic purpura patients. Medicine (Baltimore) (2019) 98(43):e17608. 10.1097/MD.0000000000017608 31651870 PMC6824755

[37] Wang Y Pang N Wang X Liu Y Wang X Wang L Percentages of PD-1(+)CD4(+)T cells and PD-L1(+)DCs are increased and sPD-1 level is elevated in patients with immune thrombocytopenia. Hum Vaccin Immunother (2018) 14(4):832–8. 10.1080/21645515.2017.1342913 PMC5893189 29333980

[38] Haanen JBAG Carbonnel F Robert C Kerr KM Peters S Larkin J Management of toxicities from immunotherapy: ESMO Clinical Practice Guidelines for diagnosis, treatment and follow-up. Ann Oncol (2017) 28(suppl_4):iv119–42. 10.1093/annonc/mdx225 28881921

[39] Brahmer JR Lacchetti C Schneider BJ Atkins MB Brassil KJ Caterino JM Management of immune-related adverse events in patients treated with immune checkpoint inhibitor therapy: American Society of Clinical Oncology Clinical Practice Guideline. J Clin Oncol (2018) 36(17):1714–68. 10.1200/JCO.2017.77.6385 PMC6481621 29442540

[40] Puzanov I Diab A Abdallah K Bingham CO 3rd Brogdon C Dadu R Managing toxicities associated with immune checkpoint inhibitors: consensus recommendations from the Society for Immunotherapy of Cancer (SITC) Toxicity Management Working Group. J Immunother Cancer (2017) 5(1):95. 10.1186/s40425-017-0300-z 29162153 PMC5697162

[41] Baldini E Lunghi A Cortesi E Turci D Signorelli D Stati V Immune-related adverse events correlate with clinical outcomes in NSCLC patients treated with nivolumab: The Italian NSCLC expanded access program. Lung Cancer (2020) 140:59–64. 10.1016/j.lungcan.2019.12.014 31881412

[42] Akamatsu H Murakami E Oyanagi J Shibaki R Kaki T Takase E Immune-related adverse events by immune checkpoint inhibitors significantly predict durable efficacy even in responders with advanced non-small cell lung cancer. Oncologist (2019) 25(4):e679–83. 10.1634/theoncologist.2019-0299 32297443 PMC7160399

[43] Das S Johnson DB Immune-related adverse events and anti-tumor efficacy of immune checkpoint inhibitors. J Immunother Cancer (2019) 7(1):306. 10.1186/s40425-019-0805-8 31730012 PMC6858629

[44] Rogado J Sánchez-Torres JM Romero-Laorden N Ballesteros AI Pacheco-Barcia V Ramos-Leví A Immune-related adverse events predict the therapeutic efficacy of anti-PD-1 antibodies in cancer patients. Eur J Cancer (2019) 109:21–7. 10.1016/j.ejca.2018.10.014 30682533

[45] Kotwal A Kottschade L Ryder M PD-L1 Inhibitor-induced thyroiditis is associated with better overall survival in cancer patients. Thyroid (2020) 30(2):177–84. 10.1089/thy.2019.0250 PMC7047075 31813343

[46] Aster RH Curtis BR McFarland JG Bougie DW Drug-induced immune thrombocytopenia: pathogenesis, diagnosis, and management. J Thromb Haemost (2009) 7(6):911–8. 10.1111/j.1538-7836.2009.03360.x PMC2935185 19344362

[47] Quirk SK Shure AK Agrawal DK Immune-mediated adverse events of anticytotoxic T lymphocyte-associated antigen 4 antibody therapy in metastatic melanoma. Transl Res (2015) 166(5):412–24. 10.1016/j.trsl.2015.06.005 PMC4609598 26118951

